# Extreme wind events responsible for an outsized role in shelf-basin exchange around the southern tip of Greenland

**DOI:** 10.1126/sciadv.adp9266

**Published:** 2024-11-13

**Authors:** Arthur Coquereau, Nicholas P. Foukal, Kjetil Våge

**Affiliations:** ^1^ Laboratoire d’Océanographie Physique et Spatiale, Univ. Brest, CNRS, IRD, Ifremer, IUEM, Brest, France.; ^2^ Woods Hole Oceanographic Institution, Woods Hole, MA 02543, USA.; ^3^ Skidaway Institute of Oceanography, University of Georgia, Savannah, GA 31411, USA.; ^4^ Geophysical Institute, University of Bergen and Bjerknes Centre for Climate Research, Bergen, Norway.

## Abstract

The coastal circulation around Southern Greenland transports fresh, buoyant water masses from the Arctic and Greenland Ice Sheet near regions of convection, sinking, and deep-water formation in the Irminger and Labrador Seas. Here, we track the pathways and fate of these fresh water masses by initializing synthetic particles in the East Greenland Coastal Current on the Southeast Greenland shelf and running them through altimetry-derived surface currents from 1993 to 2021. We report that the majority of waters (83%) remain on the shelf around the southern tip of Greenland. Variability in the shelf-basin exchange of the remaining particles closely follows the number of tip jet wind events on seasonal and interannual timescales. The probability of a particle exiting the shelf increases almost fivefold during a tip jet event. These results indicate that the number of tip jets is a close proxy of the shelf-basin exchange around Southern Greenland.

## Teaser

Particle trajectories around Southern Greenland show that shelf-to-ocean water transport is driven by extreme wind events.

## INTRODUCTION

The East Greenland Coastal Current (EGCC) is a strong, jet-like feature on the Southeast Greenland shelf that is supported by buoyant outflow from the Arctic and Greenland Ice Sheet, a strong shelf-to-coast bathymetric gradient, and northerly barrier winds, resulting from the interaction between stable air masses and the steep topography of East Greenland (*1*, *2*). The speed of the EGCC reaches over 1 m s^−1^, its volume transport is close to 1 Sverdrup (1 Sverdrup = 10^6^ m^3^ s^−1^), and its freshwater transport is a major component of the total meridional freshwater transport calculated across the Overturning in the Subpolar North Atlantic Program line (*2*, *3*). Whether this fresh water remains on the shelf around Cape Farewell at the southern tip of Greenland is critical to determining its potential effect on the Atlantic Meridional Overturning Circulation (AMOC). If transported offshore, then it has the potential to cap the warm, subtropical origin waters from giving off their heat to the atmosphere thereby reducing the water mass transformation that is essential to the subpolar AMOC (*4*, *5*). Wind plays an essential role in upper ocean currents, notably through a mechanism known as Ekman transport. This mechanism drives oceanic transport in the upper layer to the right of wind direction in the Northern Hemisphere. Around Southern Greenland, the dominant winds shift from northeasterlies along the east coast of Greenland, which induce onshore Ekman transport, to westerlies west of Greenland, which induce offshore Ekman transport. Thus, it is natural to speculate that Cape Farewell could be a region of substantial shelf-basin exchange. The fate of the EGCC waters around Cape Farewell has been discussed in many recent observational (*6*–*12*), remote sensing (*13*–*16*), and modeling studies (*17*–*31*). Yet, a consensus has yet to emerge; many studies demonstrate that much of the water is retained on the shelf [e.g., (*6*, *32*)] likely by strong potential and relative vorticity gradients across the boundary currents, while other studies have demonstrated that the fresh water is progressively removed from the Southwest Greenland shelf via eddies and meanders (*27*, *33*, *34*), as well as topographically steered flow and wind-driven transport (*6*, *10*, *26*). Whether wind-driven or eddy-induced is more important to the total remains unclear, although it is likely that both mechanisms are at work. In addition to forcing from the mean winds, the southern tip of Greenland experiences strong wind variability, with some of the strongest winds in the world ocean occurring here (*35*). In particular, tip jets (TJs) are westerly gale force winds (greater than 17 m s^−1^) to the southeast of Cape Farewell that frequently occur in winter (*36*–*38*). They occur when low pressure systems propagate from the Labrador Sea and interact with the steep topography of Southern Greenland. The westerly winds during these events are favorable to upwelling and an across-shelf offshore displacement of coastal water masses.

Determining the fate of the EGCC waters is inherently a Lagrangian problem; thus, we run Lagrangian trajectories through satellite-derived surface currents from 1993 to 2021 to explore the seasonal and interannual variability of shelf-basin exchanges around Cape Farewell. We expand on previous work (*16*) that demonstrated that the altimetry-derived surface currents (ADSC) closely mimicked in situ surface drifters on the South Greenland shelf to explore the time variability of the coastal circulation over 29 years. Here, we explore the impact of wind conditions on these exchanges. From this analysis, we discover a tight linkage between the amount of shelf-basin exchange and the number of TJs over that same time period. These results underline the crucial role played by Ekman wind–driven transport for shelf-basin exchanges.

## RESULTS

### Hotspots of shelf-basin exchanges

The simulated particles in the ADSC wrap around Cape Farewell and continue northward on the West Greenland Shelf. At the end of their passage around Cape Farewell, once west of 47°W, 83% of the initialized particles remain on the shelf (inshore of the 600-m isobath). Among the 17% of particles exported across the 600-m isobath, 14% were also exported across the 1000-m isobath, 11% across the 1500-m isobath, and 3% across the 2500-m isobath.

**Fig. 1.**
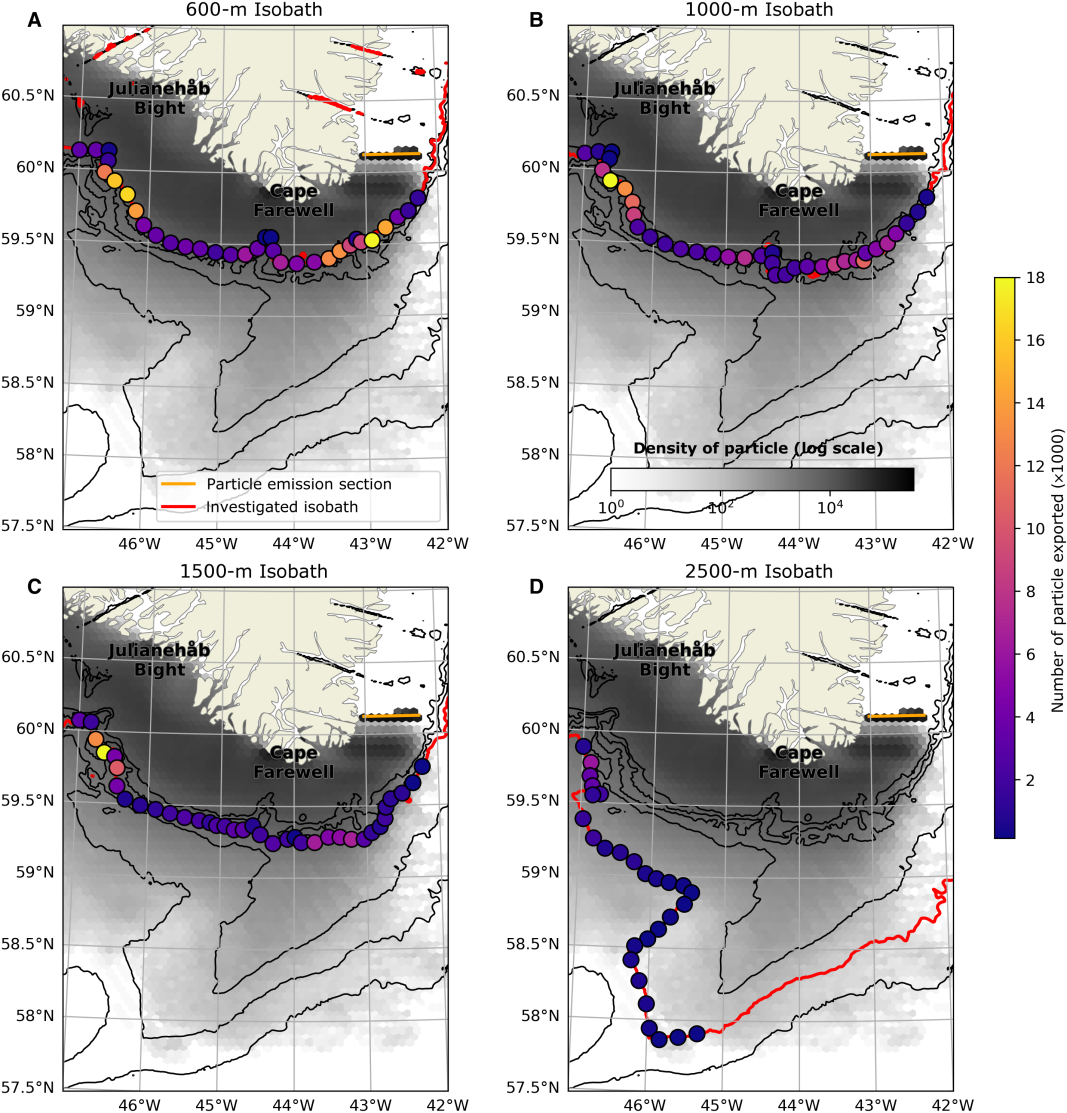
Localization of particle export for four different isobaths. Each subplot presents the results for a given isobath, highlighted in red: 600 m (**A**), 1000 m (**B**), 1500 m (**C**), and 2500 m (**D**). The gray color scale shows the density of particles over the entire experiment from 1993 to 2021, in log scale. The dots filled with the purple-to-yellow color scale represent how many particles exit the shelf at a given isobath locally. Locations where fewer than 100 particles exited during the experiment are not displayed. The zonal orange line shows the section where the particles were initialized. The black lines show the isobaths every 500 to 3000 m.

The maps of particle export locations, presented in Fig. 1, depicts two major spots of shelf-to-basin export. To the southeast of Cape Farewell, particles are exported across the 600 and 1000-m isobaths, with a few also exiting the 1500-m isobath. Almost no particles exited the 2500-m isobath here. Another hotspot appears to the west of Cape Farewell and to the south of Julianehåb Bight. Here, the shelfbreak is steeper, and this different topography allows particles to exit across all four isobaths. This spatial pattern of export demonstrates that some of the EGCC waters join the shelfbreak East Greenland Current (EGC) before rounding Cape Farewell and then continue in the boundary current to the Julianehåb Bight. This convergence between the fresh water from the shelf and the saltier water from the EGC could affect the salinity of the EGC which could consequently affect convection and water mass transformation in the boundary current [e.g., (*5*, *39*, *40*)]. A large canyon south of Cape Farewell [also detected in (*6*)] appears to have a small impact on particle exports across the 600- and 1000-m isobath, but the quantities are much smaller than those of the two hotspots. Cape Farewell therefore appears to be an important region where some of the coastal current merges into the shelfbreak jet and later leaks into the deep interior from the West Greenland shelf.

### Influence of wind conditions on particle exports

To determine why the particles exit the shelf and thus cross potential vorticity contours, which generally act as a barrier preventing shelf-basin exchanges, we then examine the time variability of the particle exports and their relation to wind forcing. We hypothesize that the number of particles exported offshore would be dictated by specific wind conditions and hence atmospheric pressure systems. In particular, westerly TJs are likely candidates for driving shelf-basin exchange in the region due to their strength and wind direction favorable to offshore Ekman transport.

**Fig. 2.**
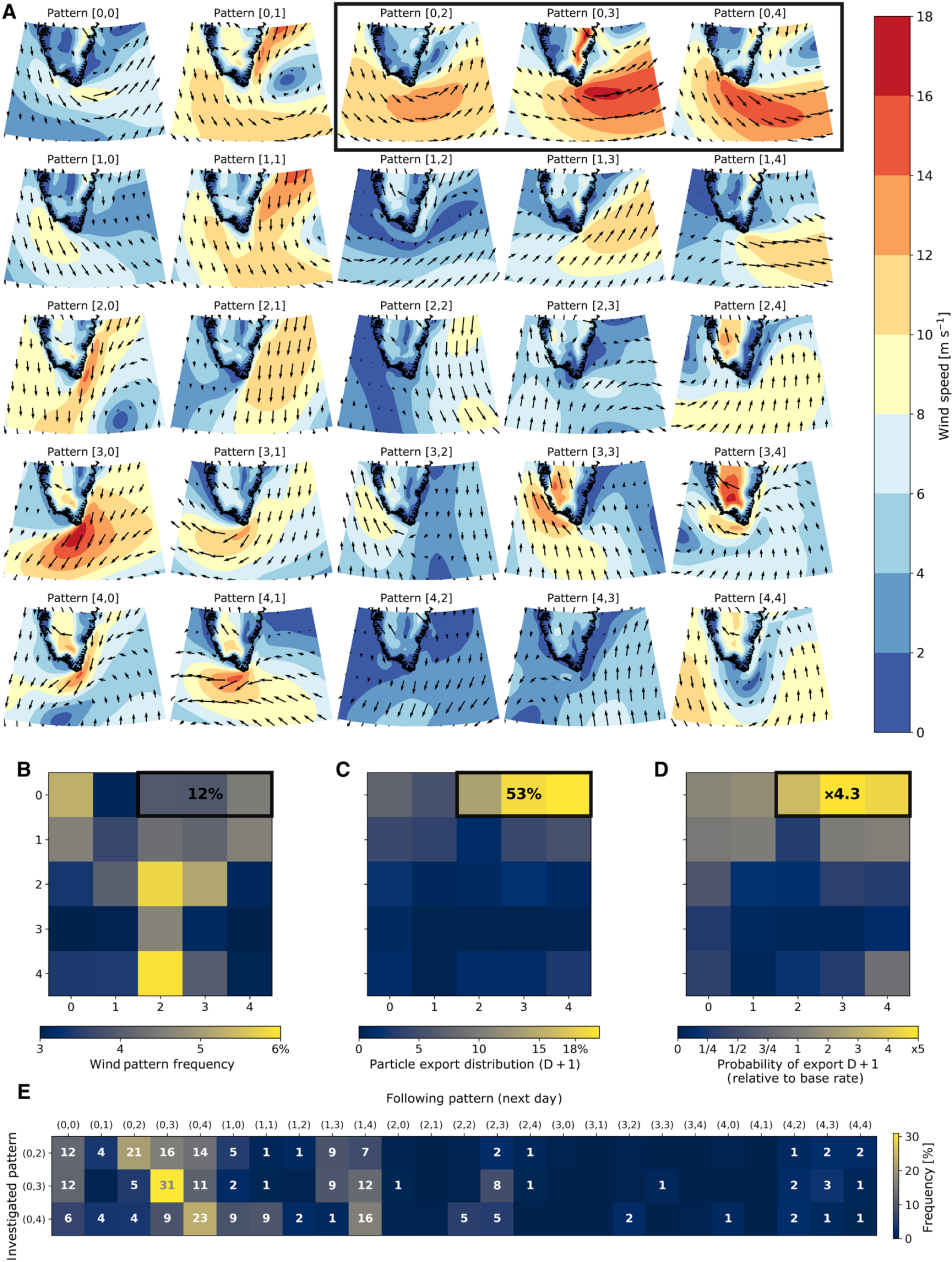
Impact of wind conditions on particle exports. (**A**) Self-Organizing Map of ERA5 wind data. (**B**) Frequency of the different wind patterns. (**C**) Number of particles exported across the 600-m isobath with each wind pattern. (**D**) Probability for a particle to be exported from the shelf when observing a given wind condition. A 1-day time lag is used for the computation of exports associated with wind events. Values provided in (B) to (D) represent the means over the three TJ wind patterns (outlined in black). (**E**) Frequency of occurrence of each pattern following the three TJ patterns.

To analyze this link between wind conditions and particle export, we use a clustering algorithm known as Self-Organizing Maps [SOM; as proposed in (*38*)] to identify 25 typical wind patterns (presented in Fig. 2) without prior knowledge on wind conditions. Each pattern of the SOM is designated by its [row, column] coordinates. The algorithm then associates each day between 1 January 1993 and 31 December 2021 with one of these patterns.

**Fig. 3.**
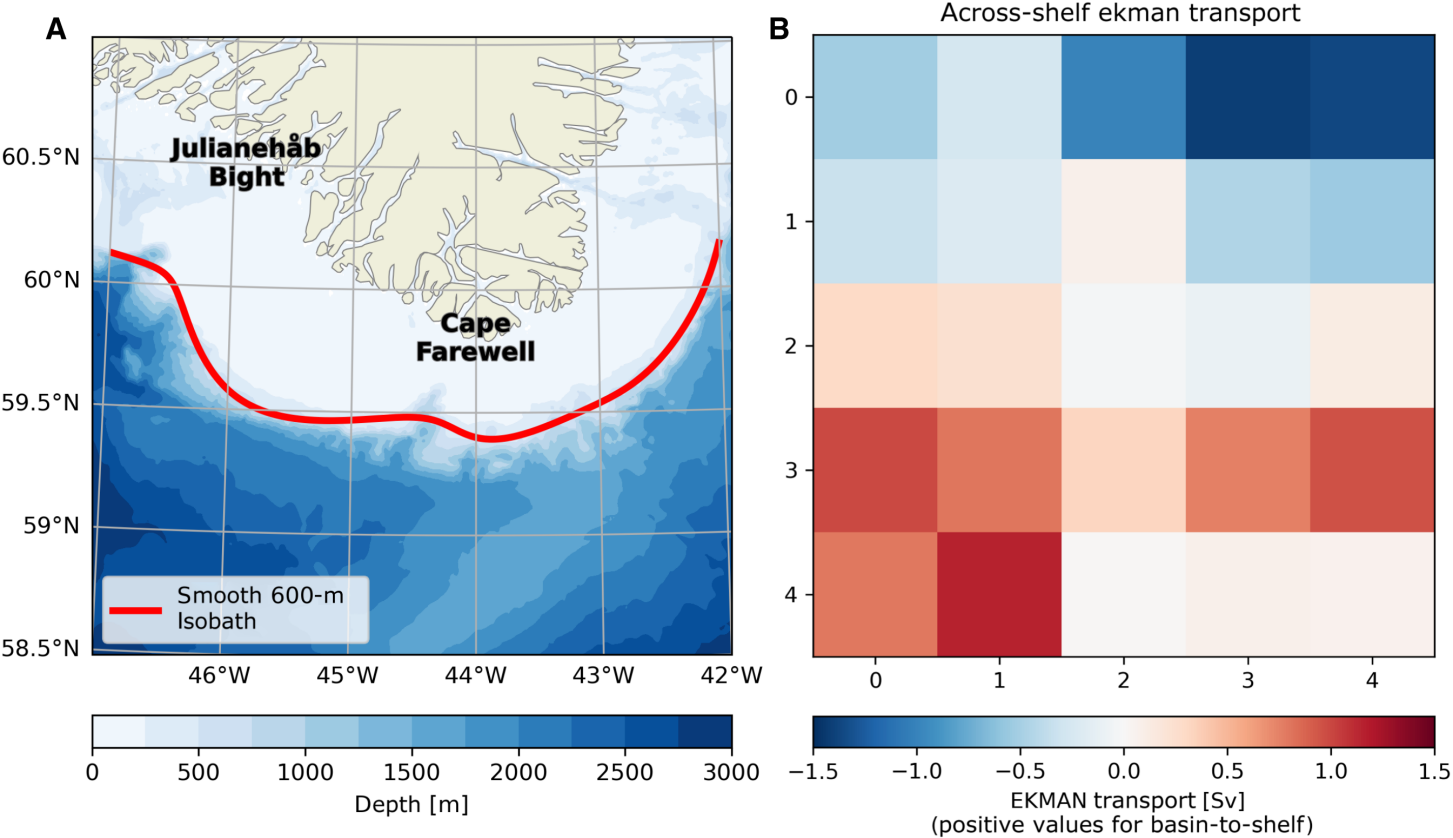
Across-shelf Ekman transport associated with wind patterns. (**A**) Map of southern tip of Greenland with the smoothed 600-m isobath (in red) across which the Ekman transport is computed. (**B**) Across-shelf Ekman transport computed across and integrated along the smooth 600-m isobath (A) around Cape Farewell for the different wind patterns derived from the SOM. The wind conditions are arranged in the same five-by-five matrix as presented in Fig. 2. Sv, Sverdrup.

The overall ability of the different patterns to drive shelf-basin exchange has been estimated from the across-shelf Ekman transport integrated along the 600-m isobath around Cape Farewell (Fig. 3). The results (Fig. 3B) provide a clear separation between two sets of conditions. On the one hand, there are patterns favorable to shelf-water export with strong negative Ekman transports associated with westerly winds (patterns [0,2], [0,3], and [0,4]). These conditions appear very similar to TJ, as described in the literature (*37*), with typical westerly winds intensified around Cape Farewell. On the other hand, there are patterns favorable to onshore currents, including patterns close to typical reverse (easterly) TJs (patterns [3,0], [3,1], [4,0], and [4,1]) and other easterly patterns ([3,3] and [3,4]).

Among the three typical TJ patterns (highlighted in black in Fig. 2), the pattern [0,3] is particularly close to the pattern depicted in (*37*), with westerly winds intensified above 16 m s^−1^ to the southeast of Cape Farewell. This specific pattern presents an average occurrence of about 14 days per year. This compares favorably with the average of 13 days of TJ per year between 1957 and 2002 reported in previous work (*37*). The two other TJ patterns show similar spatial structure and wind direction but lower intensity than the canonical TJ event. These three TJ configurations appear as the temporal evolution of an atmospheric perturbation and a progressive eastward shift of the low-pressure system. Figure 2E shows that the pattern [0,2], where the low is directly against the east coast of Greenland, is often followed by the pattern [0,3], where the low is located farther north and offshore. This pattern is then followed by pattern [0,0] or [1,4] when the low is moving rapidly eastward or decreasing substantially in intensity, or alternatively followed by pattern [0,4] if wind activity remains strong. The [1,4] pattern, where the low is far to the east, frequently follows the third TJ pattern ([0,4]). These results support the physical meaning of these patterns, which can be seen as an early, middle, and late evolution of TJs. The fact that each TJ pattern is primarily followed by itself illustrates that these wind conditions typically last longer than a day in the region and have time to develop and affect the ocean surface velocities [e.g., (*10*)].

For the computation of particle exports during a given wind event, we considered the lag between the local wind conditions and the cross-isobath movement of the particles. Previous work in the West Greenland Current shows that the Ekman effect on the local surface ocean velocities develops with a 6- to 9-hour lag from the local winds (*10*). As the location of particles are saved at daily resolution (for data storage economy), this time lag is below the frequency of the particle dataset. However, we observed that the maximum of export is lagged by 1 day after TJ conditions at daily resolution (fig. S1A). We therefore applied this 1-day lag for the computation of number of particles exported and probability of export associated with the different wind patterns (probabilities with a zero time lag are also shown in the fig. S1B). The three TJ patterns only occur between 3.9 and 4.5% of the time individually and 12% in total. However, they co-occur with 53% of the particle exports (19% for the typical TJ pattern [0,3], 20% for the pattern [0,4], and 14% for pattern [0,2]).

The average daily probability of export across the 600-m isobath, estimated to be 1.42% from the 29-year time period, is very sensitive to wind conditions (Fig. 2C). During intense TJ (pattern [0,3]), this probability is a fivefold increase from the mean (×4.9), and wind conditions presented in pattern [0,2] and [0,4] yield, respectively, a 3.5 and 4.5 times increase from the mean state. The TJ events therefore appear strongly dominant for the shelf-basin exchanges compared to other wind conditions. In contrast, the reverse TJs (patterns [3,0], [3,1], [4,0], and [4,1]) (*36*) yield a fivefold decrease compared to the mean probability of export. More generally, the non-westerlies conditions, the three bottom lines of the SOM, are observed 58% of the time and only responsible for 15% of the exports, corresponding to a fourfold decrease relative to the mean probability. This weak but nonzero probability of export is potentially due to the succession of some of these non-westerlies patterns after the TJs, as shown in Fig. 2E (e.g., for patterns [2,3] and [4,3]).

Three other patterns ([0,0], [1,3], and [1,4]) have similar spatial structure and wind direction as TJs but are weaker in magnitude (<12 m s^−1^). They are observed 14.0% of the time and responsible for 17.0% of the exports.

The probability of export relative to the base rate quantifies the ability of a given pattern to export particles, regardless of its frequency. We can compare the patterns [0,3] and [0,4], which show similar wind magnitude with spatially displaced maxima. The [0,3] is the typical TJ with westerly wind at Cape Farewell and a maximum located to the southeast of Cape Farewell. The [0,4] shows northwesterlies, with the maximum located all around Cape Farewell from the southeast to the west up to Cape Desolation. While the second pattern encompasses a large coastal region to the west of Cape Farewell and both patterns show similar velocities at the coast, the first pattern shows a greater ability to generate export. It therefore seems to indicate that the typical TJ pattern with the wind intensification to the southeast of Cape Farewell is the key atmospheric driver for shelf-basin exchanges. We further investigated these two TJ patterns by analyzing the location of exports associated with them (fig. S2). The typical TJ (pattern [0,3]) and the other eastern-intensified TJ patterns ([1,3], [1,4], and [0,0]) induce the strongest offshore flow upstream of Cape Farewell, with 62% of exports upstream of Cape Farewell for the typical TJ. In contrast, the southwestern-intensified TJ (pattern [0,4] or [1,0]) induces more offshore flow downstream of Cape Farewell (e.g., 61% for [0,4]). Despite these differences of location, the seasonal and interannual cycles remain relatively similar up- and downstream of Cape Farewell.

### Temporal variability of shelf-basin exchanges

**Fig. 4.**
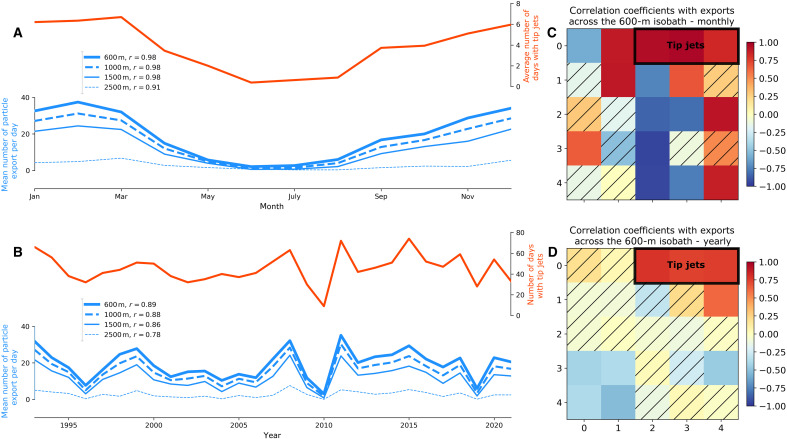
Temporal variability of particle exports and wind patterns. Comparison between the number of particles exported across a given isobath and the number of TJ events on seasonal (**A**) and interannual (**B**) timescales. (A) Mean monthly climatology of number of TJs (red, right *y* axis) and number of particle exports per day (blue, left *y* axis). (B) Annual mean number of TJs (red, right *y* axis) and number of particles exported per day (blue, left *y* axis). (**C**) Temporal correlations between the mean monthly climatology of particles exported across the 600-m isobath and the occurrence of the different wind conditions. The wind conditions are arranged in the same five-by-five matrix as presented in Fig. 2. The three TJ patterns are outlined in black (top right corner of matrix). Patterns for which the correlation coefficient is nonsignificant (outside the 95% confidence interval) are hatched in black. (**D**) Same as (C) but on interannual timescales.

We then investigated the seasonality and interannual variability of the wind patterns and their correlation with the temporal variability of the particle exports. The seasonal cycle (Fig. 4A) shows intensified offshore flow from late fall to early spring and reduced offshore flow in summer between May and August. There is also a slight bump in September, which is likely due to the peak of tropical storm season in the North Atlantic. This seasonality correlates closely to a range of wind conditions, including not only the forward (westerly) TJs (Fig. 4C) but also reverse TJs, and barrier winds (patterns [0,1], [1,1], and [2,0]) (*41*). Quiescent wind conditions that are more prevalent in summer (patterns [2,2], [3,2], and [4,2]) correlate negatively to this seasonality.

These summer conditions have almost no impact on the offshore flow (Fig. 2C). This is due to the fact that the winds are weak and oriented either perpendicular to the shelfbreak or favorable to onshore currents. The only summer pattern that shows a small export is pattern [0,0], which is essentially a very weak TJ pattern.

The seasonal variability of the three TJ patterns combined (Fig. 4A, red curve) is strongly correlated (*r* = 0.98) with the number of particles exported across the 600-m isobath, and this correlation remains unchanged if the 1000- or 1500-m isobath is considered, although the correlation with the 2500-m isobath is slightly weaker (*r* = 0.91). These results imply an enhanced exchange between the coastal current and shelfbreak current in winter due to the increase in TJ events.

The interannual time series (Fig. 4B) shows strong variability from year to year, especially from 2008 to 2011. The correlation of the wind patterns to this interannual cycle (Fig. 4D) isolates the three TJ patterns from the rest of the patterns. The correlation of these three patterns is 0.89 with the number of particles exported across the 600-m isobath and slightly lower for the 1000-m (*r* = 0.88) and 1500-m (*r* = 0.86) isobaths. Again, the correlation is lower for the 2500-m isobath but still significant with coefficient of 0.78 (and *P* value below 10^−5^).

The weak TJ in pattern [1,4] also shows a marked correlation (*r* = 0.61). This is likely due to its similar wind direction and the fact that this pattern often follows the two strongest TJ patterns ([0,3] and [0,4]) and is thus more frequent the years where many TJs occur. Most of the non-TJ patterns show nonsignificant correlations. The only exception is four patterns showing significant anticorrelation ([3,0], [3,1], [4,0], and [4,1]), which are associated with reverse TJ conditions. This demonstrates that reverse TJs effectively prevent shelf-basin exchanges by pushing shelf waters against the coast.

Correlation with the seasonal cycle is more prone to identifying unrelated phenomena, as all processes that have a seasonal cycle with similar phasing will correlate with the particle exports, regardless of the mechanisms that connect them. It is much less likely that an unrelated physical forcing would have similar interannual variability. Therefore the high correlation to TJ patterns and a physical mechanism of offshore Ekman transport are strong evidence that the TJs force particles to cross the shelfbreak.

## DISCUSSION

In this work, we show that the majority of waters (83%) in the EGCC round Cape Farewell and continue northward on the West Greenland Shelf. This result aligns with recent results from a mooring array on the Southwest Greenland shelf (*11*), as well as satellite-derived Lagrangian pathways (*15*) and modeling work (*32*). Time variability in the limited exchange between the EGC and the shelfbreak EGCC around Cape Farewell is dominated by short perturbation events rather than the mean conditions. These perturbations correspond to TJ wind events in which low pressure surface atmospheric signals are squeezed around the prominent topography of Southern Greenland, and strong westerly winds are present across the South Greenland shelf. Although these events are relatively rare (12%), they play an outsized role in the shelf-basin exchange around the southern tip of Greenland, leading to an overall 4.3 times higher probability of a coastal current particle exiting the shelf by moving water toward the shelfbreak (fig. S3). TJs can thus bring fresh water from the EGCC into contact with saltier water from the EGC, which can ultimately lead to mixing that freshens the interior. In addition, we have also detected a direct export from the shelf to the deep ocean interior, evidenced by particles exported across the 2500-m isobath, beyond the EGC. These freshwater inflows from the shelf may prevent subtropical origin waters from reaching sufficient density to convect in the Irminger and Labrador Seas and could have an impact on the subpolar AMOC intensity (*4*, *5*). The importance of short, high wind speed events on cross-shelf exchange is also illustrated by the action of reverse TJs which, unlike westerly TJs, help to retain water on the shelf and yield a fivefold decrease of the export probability.

Another important result of our study is the correlation between the number of TJs and the number of particles exported across the shelfbreak. This indicates that the number of TJs is a good indicator for estimating exchanges between the shelf and the basin around Cape Farewell and could be used in the future to monitor freshwater exchanges in this critical region without computing Lagrangian trajectories.

A caveat to the results presented here is whether the EGCC is equally well-resolved by the gridded altimetry product around all parts of the South Greenland shelf. In previous work (*16*), we determined that the Greenland coastal current is well-resolved when it detaches from the coast to the southwest of Cape Farewell [e.g., (*6*, *11*)] but that the gridded altimetry data struggle to fully resolve it when the current is most intense and banked up next to the coast on the Southeast Greenland shelf, upstream of Cape Farewell. We thus likely underestimate the number of particles released in these periods. These events correspond most closely to the barrier wind conditions that act to retain the fresh water on the inner shelf. Thus, our central result of EGCC waters being retained on the shelf may be slightly conservative and underestimated. We do not anticipate the underrepresentation of the EGCC to affect our other result that TJs drive variability in the exchange between the EGCC and EGC, because particles from the entire shelf tend to be transported toward the shelfbreak when TJs occur. Thus, we conclude that while we believe that our central results are robust, the incorporation of higher resolution altimetry data, such as those from the Surface Water Ocean Topography mission, into the gridded altimetry products will be vital to fully constraining the circulation on the South Greenland shelf. We attempted to quantify this sensitivity by smoothing the velocity fields and rerunning the Lagrangian trajectories. These results demonstrated that smoothing 1/4° fields to 1/2° and 1° yielded almost no change in the number of particles exported across the shelfbreak (fig. S4). However, whether this result holds toward higher resolution is not clear because smoothing processes that are resolved retains the underlying process, while not resolving the process initially may lead to biases. Tests in which the Ekman velocities are removed show a 10-fold reduction in the number of particle exports, underlining the importance of wind for shelf-basin exchanges, as highlighted in (*23*).

Another caveat is that we do not consider salinity in this study, which may be important to determining the freshwater exchanges between the EGC and the EGCC and ultimately between the shelf and the interior. Although the freshwater transport of the EGCC (*2*) and WGCC (*11*) is more closely connected to variability in the volume transport than the salinity of the currents, salinity still plays a role. Unfortunately, the temporal coverage of satellite-derived sea surface salinity does not extend as far back in time as altimetry, and the signal-to-noise ratio in cold waters is low. In addition, in situ measurements from moorings do not sample the upper layer above 50 m to protect against icebergs. Thus, we rely on the altimetry-derived velocities here and expect salinity to play a secondary role [e.g., (*15*)].

## MATERIALS AND METHODS

### ADSC and wind product

The velocity field used to advect the synthetic particles is the Global Total (COPERNICUS-GLOBCURRENT), Ekman and Geostrophic currents at the Surface and 15 m, acquired from the Copernicus Marine Environment Monitoring Service (CMEMS) server (https://doi.org/10.48670/mds-00327). This velocity field, with a temporal resolution of 3 hours and a spatial resolution of 1/4°, combines geostrophic surface currents derived from satellite altimetry and ageostrophic surface Ekman transport determined from European Centre for Medium-Range Weather Forecasts (ECMWF) ERA5 (*42*) wind velocity. For the geostrophic velocities, the processing used is the DUACS (Data Unification and Altimeter Combination System) multimission altimeter data processing system DT-2021 (*43*) provided by the CNES CLS (Centre National d’Études Spatiales Collecte Localisation Satellites) with a methodology detailed in (*44*). The altimetry data are merged from all available altimetry missions and interpolated on a 1/4° grid with a daily resolution. The geostrophic currents provided in this dataset use the CNES-CLS18 mean dynamic topography product (*45*) that combines altimetry, gravity, and drifter data. The geostrophic currents are computed using a nine-point stencil width methodology (*46*). The ageostrophic Ekman velocities at 15 m depth, provided by CMEMS, are computed from ERA5 (*42*) wind data following the methodology developed in (*47*–*50*). An empirical Ekman spiral-like model is estimated on the basis of two parameters determined from a least-squares regression from Surface Velocity Program (SVP) drifter data, Argo float data, and wind stress measurements from ERA5.

In previous work (*16*), we compared the same ADSC to the trajectories of 38 surface drifters deployed on the Southeast Greenland Shelf in 2021. Because of limitations in the available ship time to deploy these drifters, the drifters were restricted to a 2-week period in August and September of 2021. Over that time frame, it was demonstrated that the ADSC are able to recreate the spatial structure of the flow field and can mimic the Lagrangian nature of the flow as observed from drifters, especially close to the shelfbreak. In addition, a parallel analysis of all drifters that crossed the Southeast Greenland shelf between 1993 and 2021 from the Global Drifter Program demonstrated that the ADSC performed similarly well over those time frames. We thus have confidence that the major pathways on the South Greenland shelf are well captured by the ADSC. The detection of TJ events is based on meridional and zonal wind from the ERA5 (*42*) reanalysis with 1/4° spatial resolution and hourly temporal resolution.

### Simulation of synthetic trajectories

Particles have been initialized along a 43-km zonal line on the Southeast Greenland shelf at 60.125°N from 43.02°W to 42.25°W. Each day from 1 January 1993 to 31 December 2021, particles were initialized in proportion to the surface meridional velocity at each location along the line. We sought to release one particle for every 0.01 Sverdrup of volume transport, so we assumed a depth scale of 100 m [derived from the vertical sections presented in (*2*, *6*)] and released a particle every 100 m^2^/s of zonally integrated meridional velocity. On average, 130 particles were initialized on each day for a total of more than 1.3 million particles. The starting latitude of 60.125° provides a compromise between (i) starting sufficiently upstream of Cape Farewell to observe potential particle exports before the current rounds the southern tip of Greenland and (ii) minimizing the number of particles initialized in ice-covered cells flagged in the altimetry-dataset used for the computation of the surface currents. We ended up approximately 60 km upstream of Cape Farewell with a number of particles initialized in ice-flagged cells limited to 10% of the total number of particles.

After their initialization, the particles are advected through the surface velocity fields introduced above using the Parcels2.0 software (*51*). During the experiment, 10% of the particles were advected into the coastline. The ADSC do not conserve volume and thus may lead to convergences and divergences, especially along the coastline. The 1/4° resolution may also play a role in the beached particles by creating sharp transitions from water to land cells. We note a similar rate of beaching of observed surface drifters in the region (~10%), indicating that this onshore flow is realistic, and the beaching of particles is likely caused by the inability to represent downwelling currents. We omit these trajectories from the remaining analysis.

### Detection of particle exports

In this work, we focus on the exchanges across the shelfbreak at the southern tip of Greenland, from 42°W to 47°W. We evaluated the amount of shelf-basin exports as follows. For each particle, we detected whether it had crossed and remained off a given isobath at the end of the simulation. It is possible for a particle to cross and return to the shelf several times, in which case we retain the location and time of the particle’s last crossing.

### Wind pattern classification

We classified the wind conditions into several patterns using SOM as proposed in (*38*) using the MiniSom toolbox (*52*) in Python. The idea is to define a desired number of patterns and allow the neural network to discover the patterns and classify the high-dimensional dataset of wind conditions (every day between 1 January 1993 and 31 December 2021) onto this low-dimensional grid with patterns at each node representative of typical wind conditions. Patterns are organized in the form of a grid, with similar patterns located at nodes close to each other. Here, we examined different shapes and lastly chose a 5-by-5 map based on visual inspection that separated the major wind conditions from one another. The results were not sensitive to the shape between our 5 by 5 compared to the 7 by 5 used in (*38*). The 5-by-5 grouping yields a root mean square error of 3.59 m s^−1^.

We also compared this SOM result to the TJ detection method proposed in (*37*). That method uses Empirical Orthogonal Function (EOF) decomposition of several atmospheric properties to identify TJs. These properties have been carefully selected to see TJs emerge despite their relatively low occurrence, and they are (i) the maximum zonal wind velocity in a defined box in the southeast of Cape Farewell where TJ typically develops, (ii) the surface air temperature at this maximum velocity location, and (iii) the pressure gradients along three sections around Cape Farewell. The results obtained from the EOFs over the 1993–2021 period are consistent with results over the 1957–2002 period in (*37*) and with the SOM methodology detailed above. We decided to use SOM methodology because it appears more objective and does not require prior knowledge about TJs compared to atmospheric parameters used for EOF and the associated wind velocity threshold needed to be defined for the detection. In addition, it allows us to investigate other wind conditions beyond TJs that might play a role in driving shelf-basin exchange in the region.

### Across-shelf Ekman transport

To estimate the across-shelf Ekman transport for a given wind pattern, we start from the wind velocity field associated to the pattern and a given contour across which we want to calculate the transport. Here, the contour used is the 600-m isobath between 42° and 47°W. We then interpolate the wind field along the isobath, from west to east, and rotate the vectors, at each point along the isobath, from meridional and zonal directions to across and along shelf directions. We then use the local along-shelf wind velocity *u*_along_ to compute the along-shelf wind stress τ_along_ as

$$\tau_{\text{along}}=\rho_{\text{air}}C_{d}u_{\text{along}}|u_{\text{along}}$$ (1)

where ρ_air_ is the air density equal to 1.3 kg m^−3^ and *C*_d_ is a drag coefficient equal to 0.0015. The local across-shelf Ekman transport *U*_across_ is then computed as

$$U_{\text{across}}=\frac{-\tau_{\text{along}}}{f\;\rho_{\text{water}}}$$ (2)

where the coriolis parameter *f* = 2Ωsin(lat), Ω = 7.2921 × 10^−5^ rad/s, and ρ_water_ is the seawater density equal to 1.03 10^3^ kg m^−3^. The local across-shelf Ekman transport is then simply integrated along the 600-m isobath to obtain the total Ekman transport across the shelf associated with a given wind pattern. Positive values are associated with basin-to-shelf transport and vice versa.

### Probability of export

In addition to calculating the number of particles that leave the shelf over the course of their multiple-day trajectory, we sought to determine the probability of particles to leave the shelf on each day, which can then be compared to the wind conditions. To calculate this, we use Bayes’ theorem, which provides the probability of an event *A*, given *B*

$$p\left(A|B\right)=\frac{p\left(B|A\right)p\left(A\right)}{p\left(B\right)}$$ (3)

where the event *A* corresponds to “the particle is exported across the isobath” and the event B corresponds to “the wind pattern X is observed.” The probability of observing a given wind pattern when the particle is exported, *p*(*B*|*A*), can be computed from the number of particles exported when a given wind pattern has been detected compared to the total number of exports. We lag the particle exports by 1 day from the wind condition to account for the 6 to 9 hours it takes the wind to drive a change in the ocean velocity field [e.g., (*10*)] and the time particles take to exit the shelf.

The probability of the event *B*, *p*(*B*), is the number of days classified with a given wind pattern divided by the total number of days studied. In this work, we simplify the equation by dividing both sides by *p*(*A*). Therefore, we calculate the probability for a particle to be exported across the isobath when a given wind pattern is observed relative to the base probability of export, *p*(*A*), and is computed as

$$\frac{p\left(A|B\right)}{p\left(A\right)}=\frac{p\left(B|A\right)}{p\left(B\right)}$$ (4)

This equation produces the rate at which particles are exported across the isobath relative to the mean rate, i.e., by how much does a given wind event increase (decrease) the chance a particle is exported across the isobath? It also avoids the calculation of *p*(*A*), which is not directly available in practice, thus avoiding the inclusion of unnecessary errors. *p*(*A*) can, however, be estimated if we are interested in this base rate, assuming that the probability of a particle exiting the shelf is constant over the course of its trajectory. To do this, we first start with the overall probability of remaining on the shelf at the end of the experiment, *p*(shelf). This probability would be 100% if the duration of the trajectories is null and decreases each day by a certain amount, which corresponds to the daily probability of export *p*(*A*). To retrieve this decreasing amount, we calculate the typical duration *d* of the trajectories around Cape Farewell (e.g., the median of particle travel durations around Cape Farewell, which is 13 days). Thus, assuming that the probability is constant each day during the experiment, we then estimate the probability per day *p*(*A*)

$$p\left(A\right)=1-\sqrt[{d}]{p\left(\text{shelf}\right)}$$ (5)

This value is 1.42% for our experiment and can be interpreted as a base rate from which the values in Fig. 2D can be compared.

### Sensitivity tests

The sensitivity tests, consisting of nine additional experiments, were conducted over a 5-year period from 1 January 2014 to 31 December 2018. This period was chosen as being relatively recent (therefore expected to include good quality observations) and without extreme years for exports given the previous results. The impact of spatial and temporal resolution has been tested using two coarser spatial resolutions (1/2° and 1°) and three temporal low-pass filters (3, 7, and 14 days). The role of the Ekman contribution was investigated using the geostrophic currents provided by CMEMS. Last, we tested the impact of a stochastic forcing as proposed in *53*, to represent horizontal eddy diffusion and subgrid scale processes.

### Statistical analysis

The correlation between wind regimes and particle exports was estimated using Pearson’s correlation coefficient, and the significance of these correlations was assessed using the associated *P* values. Correlations outside the 95% confidence interval, corresponding to *P* values greater than 0.05, were highlighted in black hatches and considered nonsignificant.
