# Supplementary material for: Extreme wind events responsible for an outsized role in shelf-basin exchange around the southern tip of Greenland
Source: Sci Adv. 2024 Nov 13;10(46):eadp9266. doi: 10.1126/sciadv.adp9266 (PMC11559617; doi:10.1126/sciadv.adp9266)
Supplement: Supplementary file 1 — Figs. S1 to S4 [file sciadv.adp9266_sm.pdf]

Science Advances
AAAS

Supplementary Materials for

Extreme wind events responsible for an outsized role in shelf-basin exchange around the southern tip of Greenland

Arthur Coquereau *et al.*

Corresponding author: Arthur Coquereau, arthur.coquereau@univ-brest.fr

Sci. Adv. **10**, eadp9266 (2024)
DOI: 10.1126/sciadv.adp9266

This PDF file includes:

Figs. S1 to S4

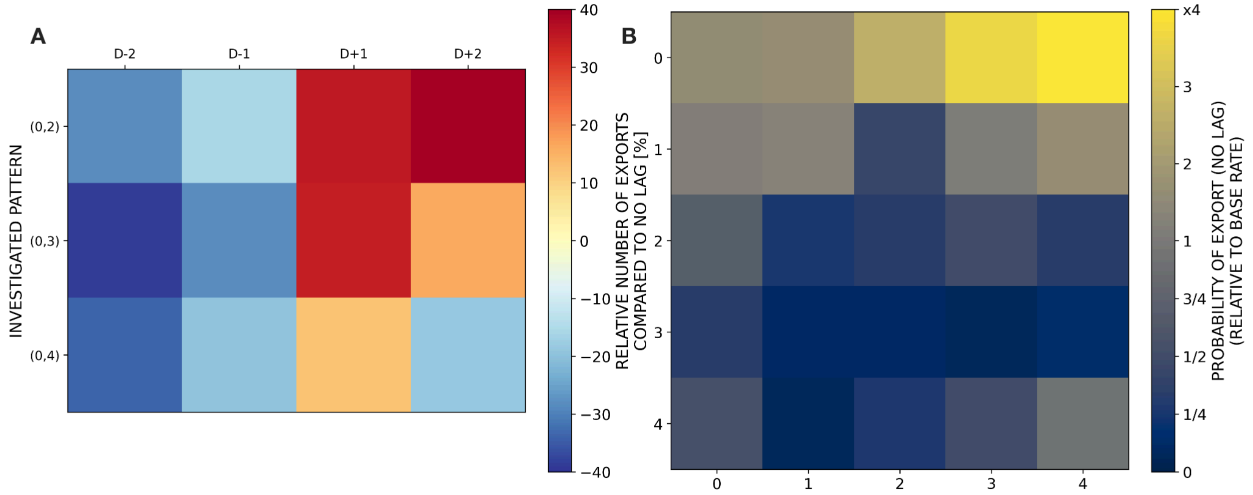

Fig. S1.

Time-lag of particle exports around a tip jet. (A). Relative number of exports for each tip jet pattern for different time-lag compared to zero lag. **(B)** Probability for a particle to be exported across the shelfbreak when observing a given wind condition compared to the base rate with zero time lag, in contrast to Figure 2 bottom right which is based on a one-day lag.

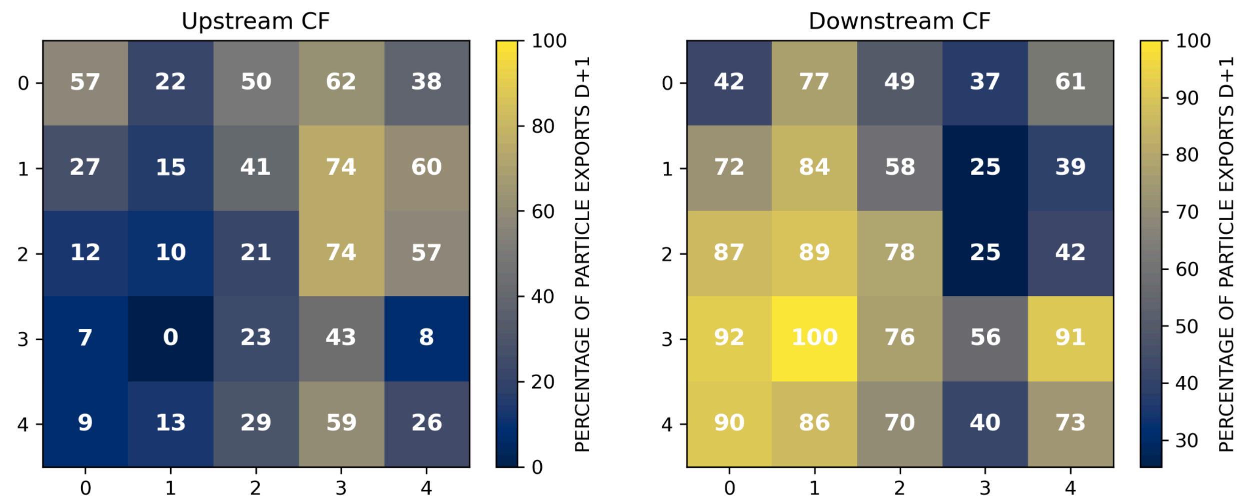

Fig. S2.

Distribution of exports upstream and downstream Cape Farewell for different wind patterns. Percentage of particle exports for each pattern (D+1) upstream and downstream Cape Farewell (CF), denoted by the 44° W meridian

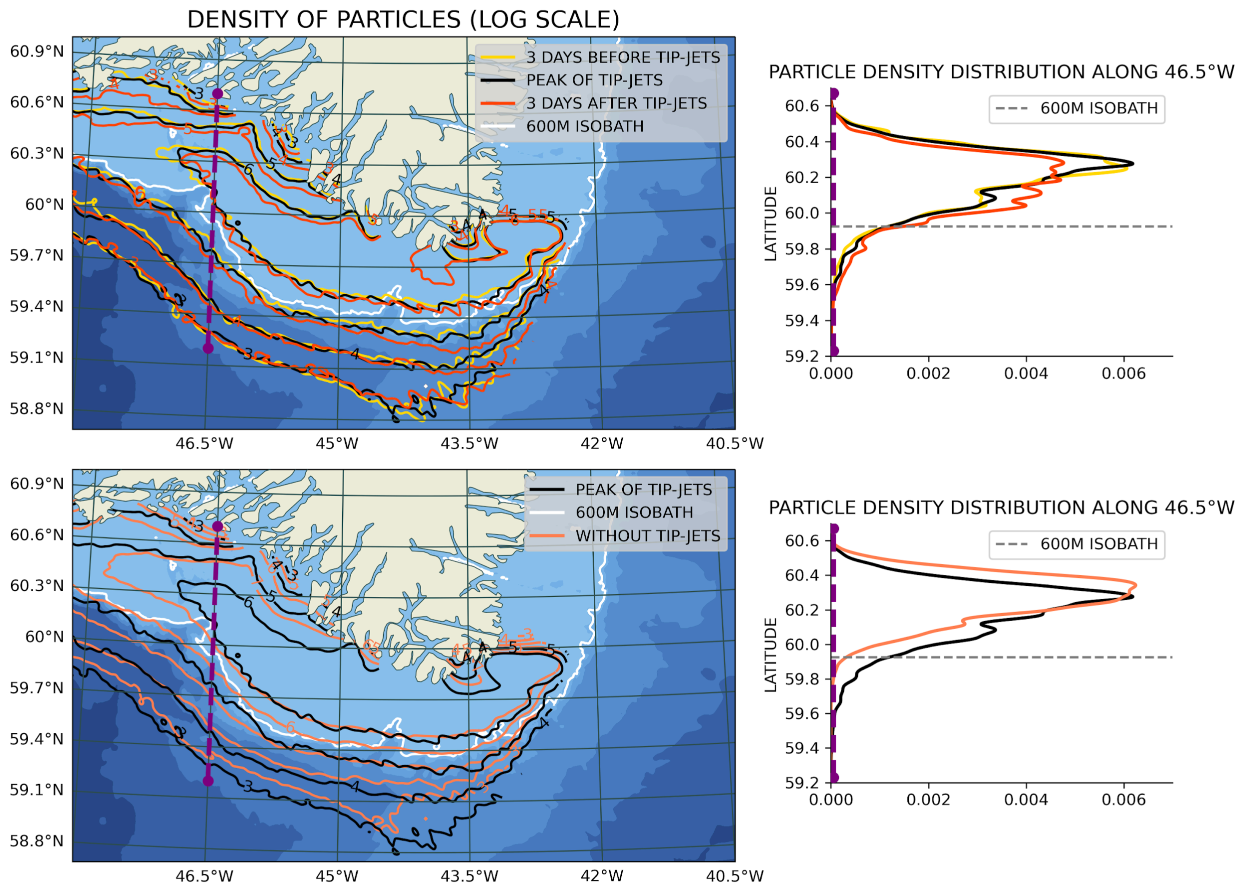

Fig. S3.

Impact of tip jets on the spatial localization of particles on the shelf. The 2D-distribution of particles in log scale is presented on the left and the distribution of particles across the shelf at 46.5°W is shown on the right panels. The top panels present the distribution of particles 3 days before, at the peak and 3 days after a tip jet. While the spatial organization of particles 3 days before and at the peak of the TJ appears very similar, the density of locations 3 days after the TJ illustrates the impact of the event, which induces an offshore shift of particles, with more particles in the outer shelf and fewer in the middle. This also shows that the effect of wind conditions takes time to manifest itself and persists several days after the event. The bottom panels present similar analysis but for tip jet or non tip jet conditions. It shows that TJ induces an offshore shift of the particles, represented by a general shift of the distribution southward. Though slight in the net spatial distribution of the particle trajectories, a small increase in the tail of the distribution that crosses the shelfbreak leads to a large signal in terms of shelf-basin exchange.

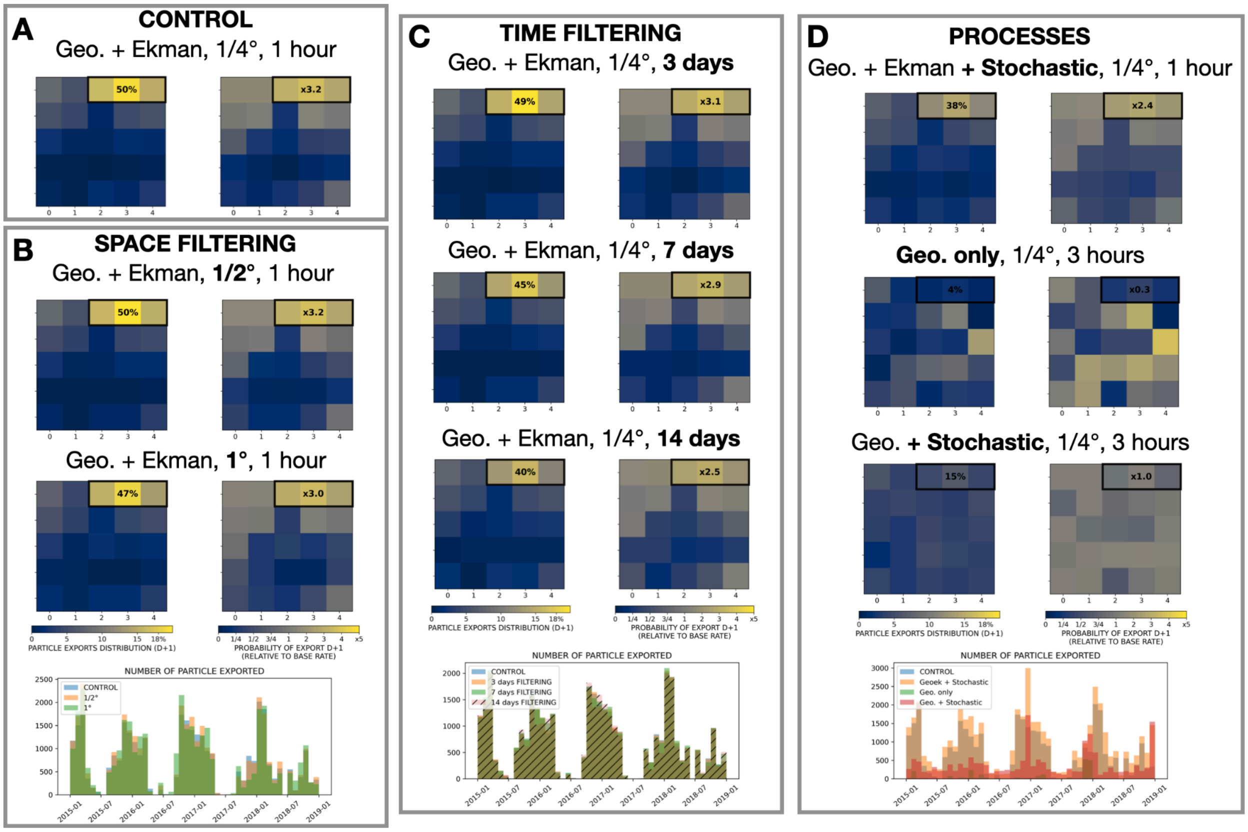

Fig. S4.

Sensitivity tests. Additional experiments with particles advected from January 1 2014 to December 31 2018. Two experiments investigate the impact of spatial (**B**) and temporal (**C**) low-pass filtering compared with a control experiment (**A**). Two spatial filterings were used: 1/2° and 1° with respect to the original 1/4° data. Three temporal filterings were investigated: 3, 7 and 14 days compared to the original hourly data. The role of different processes was also tested (**D**) in a third experiment, adding a stochastic forcing or removing Ekman contribution.
